# Lactococcal Extracellular Vesicles as In Situ Vaccine Activators in Combination with Doxorubicin for Cancer Therapy

**DOI:** 10.3390/pharmaceutics18070796

**Published:** 2026-06-28

**Authors:** Yijie Li, Chuan Chen, Yuxin Feng, Jiahe Zou, Yuqiao Qi, Weidong Huang, Yuekang Xu, Jinyao Li

**Affiliations:** 1 Xinjiang Key Laboratory of Biological Resources and Genetic Engineering, College of Life Science and Technology, Xinjiang University, Urumqi 830017, China; liyijie@xju.edu.cn (Y.L.); 107552303720@stu.xju.edu.cn (C.C.); 107552301083@stu.xju.edu.cn (Y.F.); 107552501107@stu.xju.edu.cn (J.Z.); 107552503198@stu.xju.edu.cn (Y.Q.); yuekang.xu@hotmail.com (Y.X.); 2 Xinjiang Jiayin Hospital, Urumqi 830017, China

**Keywords:** in situ vaccines, *Lactococcus lactis*, extracellular vesicles, immune activator, nanocarriers, oncotherapy

## Abstract

**Background/Objectives**: In situ vaccines that directly release endogenous tumor antigens in situ to elicit anti-tumor immune responses without exogenous antigen preparation have emerged as a promising cancer immunotherapy strategy, due to their enhanced safety by local immunization that minimizes systematically adverse reactions. However, the anti-tumor efficacy of most in situ vaccines is affected by their limited access to tumors in distant sites and the toxicity of the adjuvants contained. **Methods**: To overcome these shortcomings, the present study explored the feasibility of utilizing extracellular vesicles from the probiotic bacteria *Lactococcus lactis* as both immune activators and drug carriers, which were formulated into nanoparticles to target distant tumors. **Results**: Using confocal microscopy and flow cytometry, we confirmed that the *Lactococcus lactis*-derived extracellular vesicles possess adjuvant activity that promoted the maturation of dendritic cells without affecting their viability or apoptosis rate. Moreover, the *Lactococcus lactis*-derived extracellular vesicles, both alone and when carrying the drug doxorubicin, could target and accumulate in solid tumor tissues via the enhanced permeability and retention effect. Interestingly, compared to healthy cells, the *Lactococcus lactis*-derived extracellular vesicles tended to be taken up more by tumor cells and readily released their encapsulated doxorubicin in the acidic tumor environment, which resulted in their enhanced reactive oxygen species production and immunogenic cell death. Ultimately, systemic administration of *Lactococcus lactis*-derived extracellular vesicle-encapsulated doxorubicin greatly increased the anti-tumor efficacy by boosting the number of infiltrating dendritic cells and CD8^+^ T cells in the tumor tissues and doxorubicin-mediated immunogenic cell death. **Conclusions**: Collectively, this study demonstrated that the probiotic *Lactococcus lactis*-derived extracellular vesicles are both safer adjuvants and effective drug carriers with immunostimulatory activity and tumor-targeting capability, shedding an interesting light on this vaccine design platform for future cancer immunotherapy.

## 1. Introduction

The primary challenge in developing cancer vaccines lies in identifying tumor-specific antigens and eliciting a robust immune response [1]. Tumor antigens primarily derive from dead tumor cells, tumor cell lysates, tumor-derived exosomes, specific cancer peptides, protein antigens, and neo-antigens [1,2]. Among these, cancer vaccines targeting tumor-specific neo-antigens arising from tumor cell gene mutations have been extensively studied in recent years. However, tumor cell heterogeneity and the low frequency of neo-antigen expression have limited the efficacy of such vaccines [2]. Moreover, tumor vaccines that solely deliver tumor antigens often exhibit insufficient immunogenicity; therefore, effective cancer vaccines require immunological adjuvants and nanocarriers to enhance the immune response against tumor antigens [3]. The in situ vaccine (ISV) approach involves delivering immune activators directly into tumor tissues; this recruits and activates dendritic cells (DCs) to present endogenous tumor antigen in situ. These DCs phagocytose antigens released by immunogenic cell death (ICD) of tumor cells and present the comprehensive tumor antigens to activated T lymphocytes, thereby eliciting specific anti-tumor immunity [4]. Unlike conventional cancer vaccines delivering exogenous tumor antigens, ISVs that induce the release of endogenous tumor-associated antigens (TAAs) within the tumor microenvironment and trigger effective cytotoxic T lymphocyte (CTL) responses have emerged as a promising strategy in tumor immunotherapy. ISVs containing immune stimulants can modulate the immunosuppressive tumor microenvironment (TME), recruit and activate DCs to phagocytose antigens released by ICD, promote antigen-specific T cell-mediated tumor cell destruction, and consequently generate systemic anti-tumor immune responses [5,6]. Since systemic administration of immune adjuvants carries safety risks, current ISV vaccinations are primarily delivered intratumorally or peritumorally. However, this approach is unsuitable for repeated treatment of internal tumors or advanced visceral metastatic lesions. Although modern imaging techniques such as ultrasound, laparoscopy, and computed tomography enable precise injection into deep-seated tumors, the heterogeneity of the TME across different organs also affects the efficacy of intratumoral drug delivery [5,7,8]. Therefore, developing safer and more effective delivery methods for adjuvants that target distant tumors remains a key focus of ISV research [9].

In recent years, bacterial extracellular vesicles (BEVs), also known as bacterial membrane vesicles, have garnered significant attention due to their biomimetic properties and diverse biological functions. They are considered promising smart drug delivery systems [10] and powerful universal vaccine platforms [11]. Compared to other drug delivery systems or vaccine platforms, BEVs offer several advantages. First, they can carry a high payload of multiple active molecules and maintain these molecules in the bloodstream for extended periods, thereby enhancing bioavailability. Second, BEVs inherently contain a rich array of microbe-associated molecular patterns and possess a nanoscale membrane vesicle structure, which confers high immunogenicity. This enables them to activate both innate and adaptive immune responses [9], recruit more DCs and CD8^+^ T cells to migrate to tumor tissues, and improve immune memory in treated mice [11]. Third, the lipid spherical size of BEVs, ranging from 50 to 200 nm, allows them to passively target the solid tumor microenvironment via the enhanced permeability and retention effect (EPR effect) and to actively target tumor cells through various modifications [11,12]. Additionally, their nanoscale size facilitates their entry into lymph nodes and direct phagocytosis by lymphoid resident antigen-presenting cells (APCs) to promote adaptive immune responses [3,13]. Fourth, unlike live bacteria, BEVs are non-replicative, so that genetic engineering techniques can be employed to load exogenous proteins into the vesicle lumen or express them on the vesicle membrane surface, enabling controlled immune responses with enhanced safety [14]. Finally, BEVs are easy to produce, cost-effective, amenable to quality control, and do not face the ethical challenges associated with animal extracellular vesicle preparation [3,13,15]. Despite these advantages, however, clinical application of BEVs faces challenges such as potential biological toxicity and low natural yield. The biological toxicity primarily arises from lipid A on the surface of Gram-negative bacterial vesicles or virulence factors, including bacterial adhesins, proteases, and cytotoxins carried by pathogenic bacteria [12]. Therefore, we hypothesize that these toxic risks can be alleviated by employing Generally Recognized as Safe (GRAS) bacteria, with *Lactococcus lactis* as a typical example.

In this study, we explored BEVs derived from probiotic bacterial *Lactococcus lactis* subsp. cremoris MG1363 (LEVs), which lacks lipopolysaccharide (LPS) and virulence genes [14], and demonstrated that they could dose-dependently enhance the expression of DC surface molecules, stimulate the release of pro-inflammatory cytokines, and promote DC-mediated T cell proliferation. Furthermore, doxorubicin (DOX) loaded into LEVs (LEV-DOX) prolonged the retention time of DOX in vivo, increased cellular uptake of the drug, and facilitated tumor cell clearance through the induction of ICD and reactive oxygen species (ROS) generation, thereby inhibiting tumor growth in vivo. Importantly, intravenously administered LEV-DOX primarily accumulated in tumor tissues, with significantly lower accumulation in reticuloendothelial system organs such as the liver and spleen compared to free DOX. Moreover, LEVs did not cause damage to body weight or the heart, liver, and kidneys of mice. Instead, LEV-DOX reduced the inflammatory damage and fibrosis induced by DOX in these organs.

## 2. Materials and Methods

### 2.1. Cell Culture

The mouse monocytic macrophage leukemia cell line (RAW264.7), the mouse melanoma cell line (B16), and the rat glomerular mesangial cell line (HBZY-1) were cryopreserved at the Xinjiang Key Laboratory of Biological Resources and Genetic Engineering (Urumqi, China). RAW264.7 and HBZY-1 cells were cultured in Dulbecco’s modified Eagle medium (DMEM) supplemented with 10% fetal bovine serum (FBS), 100 U/mL penicillin, and 100 μg/mL streptomycin. B16 cells were maintained in RPMI-1640 medium supplemented with 10% FBS, 100 U/mL penicillin, and 100 μg/mL streptomycin. All cell lines were incubated at 37 °C in a humidified atmosphere containing 5% CO_2_. Bone marrow-derived dendritic cells (BM-DCs, hereinafter referred to as DCs) were generated using granulocyte-macrophage colony-stimulating factors (GM-CSF) induction, as previously described [16]. Briefly, bone marrow cells were isolated from C57BL/6J mice by syringe flushing and prepared as single-cell suspensions. These cells were then cultured in RPMI-1640 medium supplemented with 1% penicillin–streptomycin (PS), 10% FBS, 50 μmol/L β-mercaptoethanol, and 20 ng/mL recombinant GM-CSF at 37 °C under a 5% CO_2_ atmosphere. On day 2, half of the culture medium was replaced, and a complete medium change was performed on day 3. The cells were harvested on day 7 for use in subsequent experiments.

### 2.2. Synthesis and Characterization of LEVs and LEV-DOX

#### 2.2.1. Preparation for LEV

LEVs were isolated following a previously reported protocol [17], with slight modifications. *Lactococcus lactis* grew anaerobically at 30 °C in GM17 medium supplemented with 0.5% glucose until the optical density at 600 nm (OD600) reached 0.8. Ampicillin and lysozyme were then added at appropriate concentrations. After an additional 48 h of incubation, the bacterial culture was harvested and centrifuged at 10,000× *g* for 20 min at 4 °C. The supernatant was filtered through a 0.45 μm membrane and concentrated using a 100 kDa molecular weight cut-off ultrafiltration unit. The concentrate was subsequently ultracentrifuged at 150,000× *g* for 2 h at 4 °C using a 45-Ti rotor (Beckman Coulter, Indianapolis, IN, USA). The resulting pellet was washed with PBS, reconcentrated via 100 kDa ultrafiltration, and finally resuspended in PBS. Aliquots were stored at −80 °C, and fresh aliquots were used for each experiment to avoid repeated freeze–thaw cycles. The protein concentration of LEVs was determined using a bicinchoninic acid (BCA) assay kit. Vesicle morphology and size distribution were analyzed by transmission electron microscopy and dynamic light scattering.

#### 2.2.2. Preparation and Drug-Loading Efficiency of LEV-DOX

DOX was obtained from Shanghai Aladdin Biochemical Technology Co., Ltd., Shanghai, China. LEVs loaded with DOX (LEV-DOX) were prepared using a previously described optimized procedure [18]. LEVs and DOX were mixed at an optimal mass ratio in PBS and incubated with gentle stirring overnight at 37 °C. The mixture was then concentrated, and unencapsulated DOX was removed using a 100 kDa molecular weight cut-off ultrafiltration membrane. The resulting LEV-DOX was washed multiple times with PBS to ensure complete removal of any free DOX. To quantify the amount of encapsulated DOX, LEV-DOX was treated with 1% Triton X-100 for 10 min, after which absorbance was measured at 484 nm at 37 °C. Encapsulation efficiency (EE) was defined as the percentage of DOX encapsulated within LEVs relative to the total amount of DOX initially added. Drug-loading efficiency (DL) was calculated using the following equation:

DL (%) = [(Mass of initial DOX − Mass of free DOX)/(Mass of LEVs + (Mass of initial DOX − Mass of free DOX))] × 100%

#### 2.2.3. Physical Characterization

The size and surface zeta potential of the extracellular vesicle were measured using dynamic light scattering (DLS) and electrophoretic light scattering, respectively, with the Zetasizer Pro (Malvern Instruments Ltd., Malvern, UK). For transmission electron microscopy (TEM) imaging analysis of LEVs and LEV-DOX, 10 μL of purified L-EVs was applied to a carbon-coated grid for 2 min and washed twice with ddH_2_O. The sample was then negatively stained with 3% uranyl acetate for approximately 2 min and blotted dry. Electron micrographs were taken with an H7650B microscope (Hitachi, Ltd., Tokyo, Japan) at 100 kV acceleration voltage.

#### 2.2.4. In Vitro Stability and Drug-Release Profile of LEV-DOX

The in vitro release kinetics of DOX from LEV-DOX were evaluated using a dialysis-based method. Specifically, 5 mL of LEV-DOX (2 mg/mL) was placed in a dialysis bag with a molecular weight cut-off of 1000 Da and immersed in PBS (pH 6.5 or 7.4) containing 1% (*v*/*v*) Tween 80 to simulate the tumor microenvironment and systemic circulation, respectively. The assembly was incubated at 37 °C with continuous agitation for 72 h. Aliquots of the release medium were collected at predetermined time points (0, 0.5, 1, 2, 6, 12, 24, 48, and 72 h), and an equal volume of fresh, pre-warmed buffer was added after each sampling. The amount of released DOX was quantified using a standard curve generated under the corresponding pH and buffer conditions.

### 2.3. Effect of LEVs on DCs

The impact of LEVs on DCs was investigated using a method previously described in [16]. DCs were seeded at a density of 5 × 10^5^ cells per well and exposed to varying concentrations of LEVs for 12 h. Lipopolysaccharide (LPS; Sigma-Aldrich, St. Louis, MO, USA) at 20 ng/mL was used as a positive control. For phenotypic characterization, the cells were stained with fluorochrome-conjugated monoclonal antibodies specific to CD11c, CD40, CD86, MHC II, and CCR7 (labeled with PE, FITC, or APC; Elabscience Biotechnology Co., Ltd., Wuhan, China). To assess phagocytic capability, LEV- or LPS-treated DCs were incubated with 20 µg/mL FITC-labeled dextran (Sigma-Aldrich, St. Louis, MO, USA) for 12 h. The cells were then collected and analyzed using a Cyto FLEX flow cytometer (Beckman Coulter, Indianapolis, IN, USA). Data analysis was performed using Cytexpert 2.6 software (Beckman Coulter, Inc., Brea, CA, USA). Cytokine production by DCs after 12 h of LEV treatment was measured using enzyme-linked immunosorbent assay (ELISA). Supernatants were collected, and levels of IL-1β, TNF-α, IL-6, IL-12, and IL-10 were determined using commercially available ELISA kits (Boster Biological Technology Co., Ltd., Wuhan, China), following the manufacturers’ protocols.

### 2.4. Mixed Lymphocyte Reaction (MLR)

The MLR was conducted following established protocols. DCs were isolated from C57BL/6 mice and treated with LEVs for 12 h. Subsequently, 1 × 10^5^ treated DCs were washed and seeded into 24-well plates containing 0.5 mL of complete RPMI-1640 medium. Splenocytes obtained from BALB/c mice were labeled with carboxy fluorescein succinimidyl ester (CFSE; eBioscience Biotechnology Co., Ltd., Wuhan, China) and co-cultured with the pre-treated DCs at a density of 1 × 10^6^ cells in 0.5 mL of complete RPMI-1640 medium. The co-culture system was incubated for 72 h at 37 °C under 5% CO_2_. T cell proliferation was assessed by flow cytometry, with LPS-treated DCs serving as the positive control.

### 2.5. In Vivo DC Migration Assay

The migratory capacity of DCs in vivo was assessed using established methods [19]. Untreated DCs or DCs treated with LEVs (1 or 10 µg/mL) and LPS-stimulated DCs were stained with carboxyfluorescein succinimidyl ester (CFSE). A total of 1 × 10^6^ labeled cells in 50 µL of PBS were administered via subcutaneous injection into the right hind footpad of each mouse. After 24 h, both the ipsilateral and contralateral inguinal lymph nodes (LNs) were excised. DC migration was quantified by detecting CFSE^+^ cells in the lymph nodes using flow cytometric analysis.

### 2.6. Cellular Uptake of LEVs

Cellular uptake of LEVs was evaluated by labeling the vesicles with the lipophilic fluorescent tracer 3,3′-dioctadecyloxacarbocyanine perchlorate (DiD; Invitrogen, Carlsbad, CA, USA). Intracellular localization was monitored using confocal microscopy. RAW264.7, B16, or HBZY-1 cells were plated onto confocal dishes (NEST Biotechnology, Wuxi, China) and cultured overnight. The cells were then treated for 0, 2, or 6 h with one of the following: 20 μg/mL free DOX; LEV-DOX containing an equivalent DOX concentration; or LEVs at the same volume. Nuclei were counterstained with DAPI. Imaging was performed using an A1RHD25 confocal microscope (Nikon Corporation, Tokyo, Japan) equipped with dark-field and fluorescence capabilities. The following excitation/emission wavelengths were used: 488/575 nm for DOX, 644/665 nm for DiD, and 350/461 nm for DAPI.

### 2.7. In Vitro Evaluation of Cell Viability and Apoptosis

Cell viability was quantified using the tetrazolium-based MTT colorimetric assay. B16 cells were plated at a density of 5 × 10^3^ cells per well in 96-well plates and incubated overnight. The cells were then treated with free DOX, LEV-DOX, or LEVs for 8 h. Subsequently, 10 μL of MTT solution (5 mg/mL) was added to each well, followed by incubation at 37 °C for four hours. The MTT-containing medium was carefully aspirated, and the formazan crystals were solubilized with 100 μL of dimethyl sulfoxide (DMSO) per well. Absorbance was measured at 570 nm using a Varioskan LUX microplate reader (Thermo Fisher Scientific, Waltham, MA, USA), and cell viability was expressed as a percentage relative to the control group. Apoptosis was evaluated using an Annexin V-FITC/propidium iodide (PI) apoptosis detection kit (Yeasen Biotechnology (Shanghai) Co., Ltd., Shanghai, China). Briefly, B16 cells or DCs were seeded in 6-well plates and cultured at 37 °C in a 5% CO_2_ atmosphere until they reached approximately 80% confluence. The cells were then treated for 24 h with PBS (control), LEV, free DOX, or LEV-DOX at equivalent DOX concentrations (0, 3, 6, 12, 25, and 50 μg/mL). After treatment, the cells were collected, washed twice with ice-cold PBS, and stained with Annexin V-FITC and PI according to the manufacturer’s protocol. Apoptotic cells were detected and quantified by flow cytometry.

### 2.8. Establishment of the Animal Model

All experimental procedures involving animals were approved by the Institutional Animal Care and Use Committee of Xinjiang University (approval no. XJUAE-2024-042, 15 December 2024) and conducted in accordance with relevant guidelines. Female BALB/c and C57BL/6J mice (aged 4–6 weeks) were obtained from the Experimental Animal Centre of Xinjiang Medical University. The mice underwent a seven-day acclimatization period under specific pathogen-free (SPF) conditions at Xinjiang University’s animal facility. During this period, the animals were monitored daily for any signs of morphological or behavioral abnormalities. Only healthy mice were selected for subsequent experiments.

#### 2.8.1. In Vivo Biodistribution of LEV-DOX

4T1 cells (1 × 10^6^) were subcutaneously injected into the right flank of BALB/c mice. When tumor volumes reached approximately 100 mm^3^, tumor-bearing mice were randomly assigned to two groups *(n* = 3 per group). The mice received a single intravenous injection via the tail vein of either free DiR or DiR-LEV at a dose equivalent to 5 μL/g body weight of DiR. Blood samples (150 μL) were collected from the retro-orbital plexus at 0, 0.5, 2, 4, 6, 12 and 24 h post-injection. Twenty-four hours after administration, all mice were humanely euthanized, and their major organs and tumors were excised. The fluorescence intensity of DOX in plasma was measured using a microplate reader. The biodistribution of LEVs in the organs was assessed using a small-animal in vivo imaging system (FOBI Imaging System, CELLGENTEK), and the fluorescence intensity of DOX in plasma, organs, and tumors was quantitatively analyzed using Fiji (ImageJ software, 1.54f), Max Planck Institute of Molecular Cell Biology and Genetics, Dresden, Germany.

#### 2.8.2. Evaluation of Anti-Tumor Efficacy in a Subcutaneous Xenograft Model

B16 cells (1 × 10^6^) were subcutaneously injected into the right flank of BALB/c mice. When tumor volumes reached approximately 80 mm^3^, tumor-bearing mice were randomly divided into four groups (*n* = 6). The treatment groups received the following via intravenous injection: PBS, LEV, DOX, or LEV-DOX. All formulations were administered at a DOX-equivalent dose of 2 mg/kg. The treatments on the animals were performed every 3 days for a total of 4 doses. Throughout the 11-day treatment period, body weight and tumor volume were monitored and recorded every three days. Tumor volume (V) was calculated using the following formula: V (mm^3^) = length (mm) × [width (mm)]^2^/2. On day 20, blood samples were collected from the retro-orbital plexus under anesthesia. Afterwards, all mice were humanely euthanized. The major organs (heart, liver, spleen, lungs, and kidneys) and tumor tissues were excised and processed for hematoxylin and eosin (H&E) staining. To ensure animal welfare, any mouse that developed a tumor volume exceeding 1500 mm^3^ or exhibited signs of severe morbidity during the experiment was euthanized immediately.

### 2.9. Analysis of Serum Biochemical Parameters

Serum samples collected from tumor-bearing mice were subjected to biochemical analysis. Cytokine levels (IFN-γ and IL-4) were quantified using commercial ELISA kits (Elabscience, Biotechnology Co., Ltd., Wuhan, China) according to the manufacturer’s instructions. Absorbance was measured at 450 nm using a BioTek Epoch 2 microplate reader (BioTek Instruments, Inc., Santa Clara, CA, USA). Additionally, organ injury markers were evaluated using microplate-based assays with the following kits, following their respective protocols: creatine kinase (CK), lactate dehydrogenase (LDH), alanine aminotransferase (ALT/GPT), aspartate aminotransferase (AST/GOT), blood urea nitrogen (BUN), and creatinine (CRE).

### 2.10. Statistical Analysis

All data are expressed as the mean ± standard error of the mean (SEM). Statistical comparisons were performed using one-way analysis of variance (ANOVA). Data analysis was conducted using GraphPad Prism version 9 (GraphPad Software, San Diego, CA, USA). Differences were considered statistically significant at *p* < 0.05.

## 3. Results

### 3.1. Preparation and Characterization of LEVs and LEV-DOX

**Figure 1:**
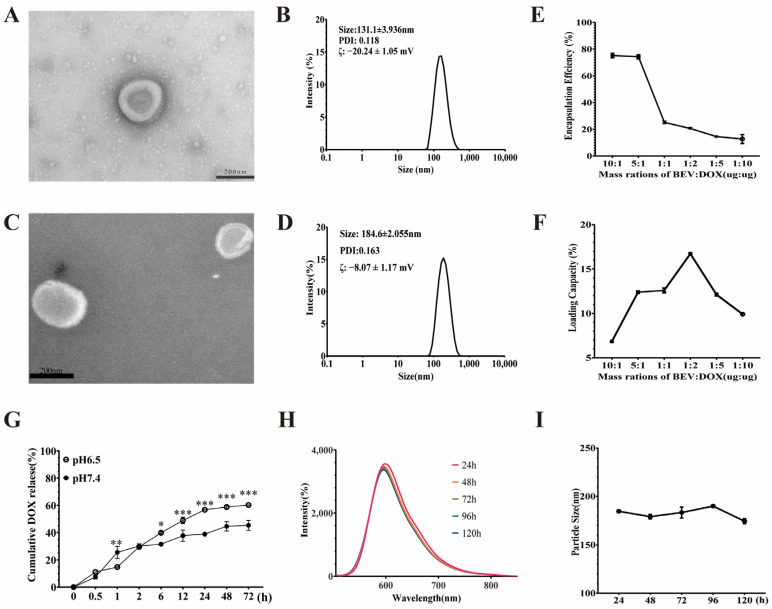
Preparation and characterization of LEVs and LEV-DOX. (**A**,**C**) Representative micrographs of LEV (**A**) and LEV-DOX (**C**) captured by TEM. The scale bars are 200 nm. (**B**,**D**) Size distributions of LEVs (**B**) and LEV-DOX (**D**) were analyzed using NTA. (**E**,**F**) The relative encapsulation efficiency (**E**) and loading efficiency (**F**) of DOX was determined. (**G**) The release efficiency of DOX from LEV-DOX at pH 6.5 and pH 7.4. (**H**). Fluorescence stability of DOX content in LEV-DOX at different time periods. (**I**) Particle size stability of LEV-DOX at different time periods. Data are from three independent experiments and analyzed by ANOVA. The error bars are shown as mean ± SEM. * *p* < 0.05; ** *p* < 0.01; and *** *p* < 0.001 compared to untreated group or control group. LEV: *Lactococcus lactis*-derived extracellular vesicle; LEV-DOX: LEV loaded with doxorubicin.

LEVs were prepared using ultracentrifugation combined with ultrafiltration. The size and uniformity of LEVs and LEV-DOX vesicles were characterized by TEM and DLS. The LEVs isolated from *Lactococcus lactis* exhibited a spherical lipid bilayer structure with an average particle size of 131.1 ± 3.94 nm and a relatively narrow size distribution (polydispersity index, PDI = 0.118). (Figure 1A,B). Subsequently, LEV-loaded DOX (LEV-DOX) was obtained by co-incubating LEVs and DOX at an appropriate ratio, followed by ultrafiltration to remove free DOX. As shown in Figure 1E,F, the encapsulation efficiency decreased significantly with decreasing LEV: DOX mass ratio, while drug loading steadily increased. For example, at a LEV: DOX mass ratio of 1:2, although the encapsulation efficiency was only 20.6%, the drug loading reached 16.7%. Particle size analysis indicated that DOX encapsulation did not affect the uniformity of LEVs; however, the average diameter of LEV-DOX increased to 184.6 ± 2.05 nm with a relatively narrow size distribution (PDI = 0.163) (Figure 1C,D). To further investigate stability according to surface charge, we measured the zeta potential of LEVs and LEV-Dox. The measured surface charge of LEVs was −20.24 ± 1.05 mV; however, it significantly increased after drug loading, with LEV-DOX measuring −8.07 ± 1.17 mV.

Since we intend to target distant tumors, we compared drug release between the cancer cell microenvironment and normal physiological conditions, by testing the LEV-DOX in their corresponding pH values (6.5 and 7.4). Interestingly, we found that LEV-DOX released relatively slowly in the physiological environment of neutral pH, but increased under acidic conditions of tumor tissues, 45.3% vs. 60.2% at 72 h). This indicates the potential of LEVs to preferentially release their encapsulated drugs in the tumor microenvironment (Figure 1G). Subsequently, we measured the fluorescence intensity and particle size changes of LEV-DOX at different time points (24, 48, 72, 96, and 120 h). The fluorescence intensity profile showed that LEV-DOX maintained overall stability, with only slight decreases in fluorescence intensity from 24 to 120 h at 37 °C, without significant fluctuations. This indicates that LEVs maintained good drug encapsulation and stability at 37 °C (Figure 1H). The particle size variation profile demonstrated that the particle size of LEVs remained around 180 nm with minimal changes at 37 °C, indicating that LEVs did not aggregate or swell over the long term in vivo and remained within an appropriate size range (Figure 1I).

### 3.2. In Vitro Activation of DCs and T Cells by LEVs

**Figure 2:**
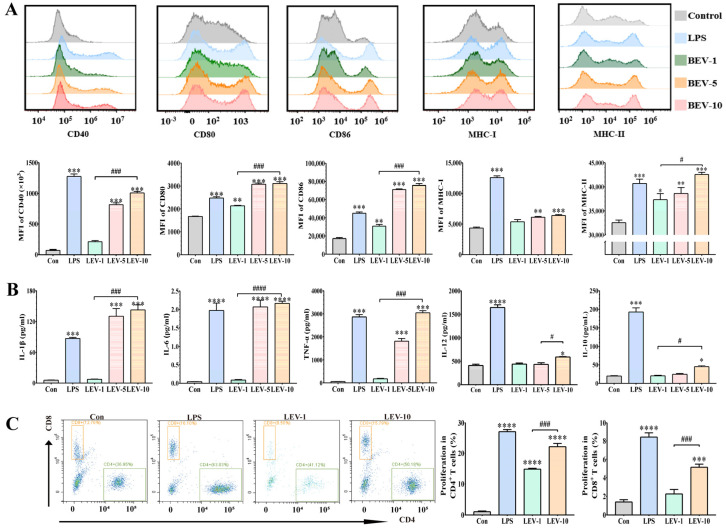
DC maturation and cytokine production after LEV treatment. DCs were induced from bone marrow of C57BL/6 mice in the presence of GM-CSF. On day 7, cells (1 × 10^6^/mL) were treated with different concentrations (1, 5 and 10 μg/mL) of LEVs for 12 h. LPS (20 ng/mL) was used as a positive control. (**A**) After treatment, the expressions of co-stimulatory molecules, MHC I and MHC II on DCs were detected by flow cytometry in upper panels. The mean fluorescence intensity (MFI) of co-stimulatory molecules, MHC I and MHC II are shown in the lower panels. (**B**) The supernatants were collected and the production of IL-1β, IL-6, IL-10, IL-12 and TNF-α was detected by ELISA. The concentrations of cytokines are shown. (**C**) MLR was performed using C57BL/6 DCs and BALB/c splenocytes. DCs on day 7 were treated with different concentrations (1 and 10 μg/mL) of LEVs or LPS for 12 h and then treated with mitomycin C. Splenocytes were obtained from BALB/c mice. DCs and splenocytes at ratios of 1:5 were co-cultured for 72 h. Cell proliferation was detected by flow cytometry. Data are from four independent experiments and analyzed by ANOVA. The error bars are shown as mean ± SEM. * *p* < 0.05; ** *p* < 0.01; *** *p* < 0.001 and **** *p* < 0.0001 compared to the untreated group or control group. # *p* < 0.05, ### *p* < 0.001 and #### *p* < 0.0001 compared with each other. LEV: *Lactococcus lactis*-derived extracellular vesicle. DCs: dendritic cells; GM-CSF: granulocyte-macrophage colony-stimulating factor; MLR: mixed lymphocyte reaction; LPS: lipopolysaccharide.

Although the probiotic LEVs lack toxins, they contain microbe-associated molecular patterns (MAMPs); therefore, we first evaluated their immunostimulatory capacity by co-incubating different concentrations of LEVs or 20 ng/mL LPS with DCs. We subsequently assessed the surface molecule expression and cytokine secretion of the DCs by flow cytometry and ELISA, respectively. Flow cytometry results showed that, compared with the untreated control group, LPS significantly increased the expression levels of all markers, validating the effectiveness of the experimental system. Among the LEV-treated groups at different concentrations, 5 µg/mL and 10 µg/mL LEVs significantly promoted the expression of DC surface molecules CD40, CD80, CD86, MHC class I, and MHC class II (*p* < 0.001) in a dose-dependent manner (Figure 2A,B). Similarly, LEVs at concentrations above 5 µg/mL also significantly stimulated DCs to secrete IL-1β, IL-6, TNF-α, IL-12 and the anti-inflammatory cytokine IL-10 in the same way as what positive control LPS did (Figure 2C). Notably, 10 µg/mL LEVs stimulated DCs to secrete both IL-12 and IL-10, with the IL-12 concentration approximately 13 times higher than that of IL-10 (Figure 2C).

**Figure A1:**
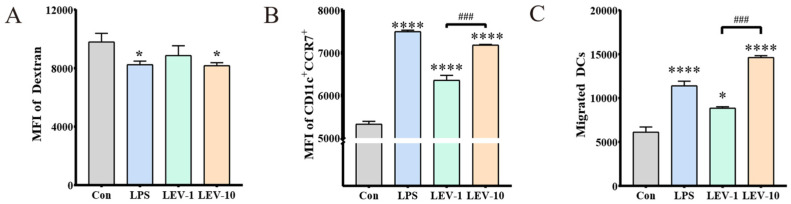
Effect of different concentrations of LEVs on DC migration ability. (**A**) Uptake of FITC-dextran by DC. (**B**) CCR7 as measured by FCM. (**C**) Transwell assay for the assessment of DC migration in vitro. Data are from three independent experiments and analyzed by ANOVA. The error bars are shown as mean ± SEM. * *p* < 0.05 and **** *p* < 0.0001 compared with the PBS group (Con); ### *p* < 0.001 compared with each other. LEV: *Lactococcus lactis*-derived extracellular vesicle.

Since mature DCs tend to reduce their antigen phagocytic activity while enhancing their ability to migrate to lymph nodes, we next examined the ability of LEV-stimulated DCs to phagocytose fluorescein isothiocyanate (FITC)-labeled dextran, the expression levels of the DC migration marker CCR7, and the number of migrated DCs in the popliteal lymph nodes after footpad injection. As shown in Figure A1A, both LPS and 10 µg/mL LEVs significantly inhibited DC phagocytic activity (*p* < 0.05). Figure A1B,C demonstrated that both LPS and LEVs at concentrations of 1 µg/mL and 10 µg/mL significantly increased the expression of CCR7 on DCs, and the number of migrated DCs in the popliteal lymph nodes. These results indicated that LEVs at concentrations above 1 µg/mL can significantly enhance CCR7 expression and promote DC migration to lymph nodes.

To assess the effect of LEVs on the ability of DCs to promote T cell proliferation following activation, DCs treated with LPS or LEVs were co-cultured with CFSE-labeled T cells from allogeneic splenocytes. As shown in Figure 2C, compared with the PBS-treated group, DCs activated with LPS significantly enhanced the proliferation of splenic CD4^+^ and CD8^+^ T cells as expected. Notably, DCs activated by LEVs at a dose of 10 µg/mL also significantly promoted the proliferation of both CD4^+^ and CD8^+^ T cells (*p* < 0.001). DCs treated with LEVs at a concentration of 1 µg/mL, however, only significantly promoted the proliferation of CD4^+^ T cells, but not CD8^+^ T cells, compared with the PBS-treated group. Nevertheless, these results indicate that LEVs exhibited strong adjuvant activity in stimulating DC maturation and T cell stimulation.

### 3.3. Effects of LEVs on Cellular Uptake and LEV-DOX-Induced Cell Killing

**Figure 3:**
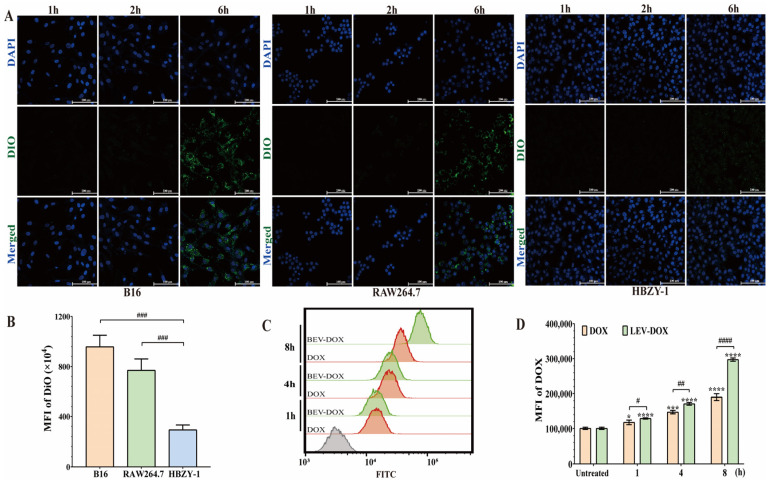
In vitro cellular uptake of DiO-labeled LEVs by different cell types. (**A**) In vitro cellular uptake of DiO-labeled LEVs by B16, RAW264.7 and HBZY-1 with the function of time. The merged images show a time-dependent increase in the intracellular accumulation of LEVs. Scale bars are 100 μm. (**B**) The MFI detected by flow cytometry after LEVS was incubated with different cells for 12 h. (**C**,**D**) Flow cytometric analysis of the temporal dynamics in B16 cell uptake of free DOX versus LEV-DOX. The image shows that the concentration of LEV-DOX ingested by B16 cells was significantly higher than that of free DOX. Data are from three independent experiments and analyzed by ANOVA. The error bars are shown as mean ± SEM. * *p* < 0.05; *** *p* < 0.001 and **** *p* < 0.0001 compared to the untreated group or control group. # *p* < 0.05, ## *p* < 0.01, ### *p* < 0.001 and #### *p* < 0.0001 compared with each other. LEV: *Lactococcus lactis*-derived extracellular vesicle; LEV-DOX: LEV loaded with doxorubicin.

One of the important features for a good in situ adjuvant is its capacity to be taken up and absorbed in the local tissues for immune activation. To compare the differences in probiotic LEV uptake by various cell types, we employed confocal laser microscopy (Figure 3A,B) to detect the internalization of DiO-labeled LEVs by rat glomerular mesangial cells (HBZY-1), mouse monocytic leukemia cells (RAW 264.7), and tumor cells (B16). Interestingly, we found that after 6 h of co-incubation, tumor cells exhibited much higher LEV uptake efficiency than that of normal somatic HBZY-1 cells, with that of macrophages falling in between (Figure 3A,B), which added great value to our probiotic LEVs for targeting tumors at a distance. Next, we assessed the effect of LEVs on carrying and delivering DOX to tumor cells. B16 cells were co-incubated with either free DOX or LEV-encapsulated DOX (LEV-DOX) for varying durations, and intracellular DOX fluorescence intensity was measured by flow cytometry. As shown in Figure 3C,D, after 1, 4, and 8 h of co-incubation at equivalent DOX concentrations, flow cytometry revealed a significant, time-dependent increase in intracellular DOX fluorescence signals. Notably, as early as 1 h, the mean fluorescence intensity of DOX in B16 cells treated with LEV-DOX was significantly higher than in cells treated with free DOX, indicating that LEVs, as a drug delivery vehicle, greatly enhanced the uptake of DOX by tumor cells within the observed time frame.

**Figure 4:**
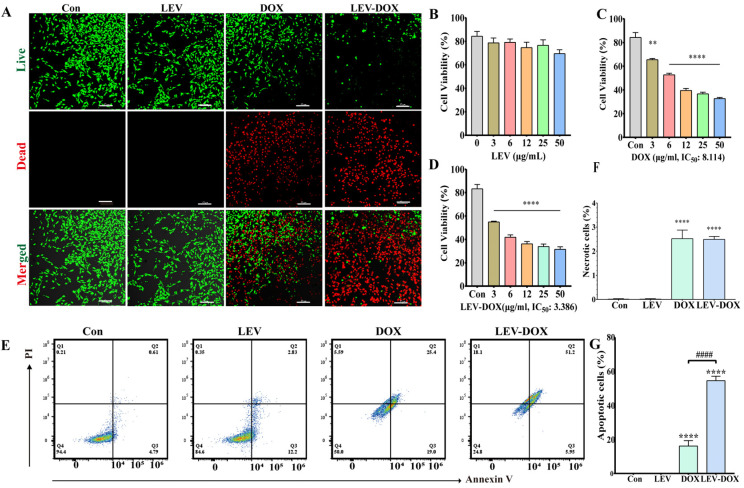
Evaluation of cell death, viability and apoptosis levels in B16 cells following 24 h treatment with LEVs, free DOX or LEV-DOX. (**A**) Calcein-AM/PI confirmed the death level of LEV-, free DOX- or LEV-DOX-stimulated B16 cells. The living cells stained with Calcein-AM are marked with green fluorescence, and the dead cells stained with PI are marked with red fluorescence. Scale bars are 100 μm. (**B**–**D**) B16 cells were incubated with LEVs (**B**), free DOX (**C**) or LEV-DOX (**D**) (0, 3, 6, 12, 25 and 50 μg/mL) for 24 h; cells were collected, and cell viability was measured by MTT assay. (**E**–**G**) B16 cells were incubated with LEV, free DOX or LEV-DOX at the indicated concentrations for 24 h, and cell necrosis or apoptosis was analyzed by flow cytometry. Data are from three independent experiments and analyzed by ANOVA. The error bars shown as mean ± SEM. ** *p* < 0.01; and **** *p* < 0.0001 compared to the untreated group or control group. #### *p* < 0.0001 compared with each other. LEV: *Lactococcus lactis*-derived extracellular vesicle; LEV-DOX: LEV loaded with doxorubicin.

To evaluate the effects of tumor toxicity delivered by the LEV-encapsulated DOX, the viability and apoptosis of B16 cells treated with LEVs and LEV-DOX were assessed using MTT, PI/AM, and Annexin V/PI staining assays. As shown in Figure 4A, in both the PBS-treated group and the group treated with 50 µg/mL LEVs, most B16 cells exhibited healthy green fluorescence indicative of alive cells. Furthermore, this observation was supported by the MTT assay results (Figure 4B). In contrast, the group treated with 3 µg/mL DOX alone showed a substantial increase in red fluorescence, indicating significant cell death and the effectiveness of this cell-toxic drug. Interestingly, compared with the DOX alone, LEV-DOX at the same DOX concentration resulted in a higher cell death rate, indicating more drug substances were delivered to the tumor cells by the bacterial extracellular vesicles. Moreover, this enhanced toxic effect by LEV-DOX was further corroborated by the MTT results (Figure 4C,D). Further analysis of the drug-induced cell death (Figure 4E–G) revealed that LEV-DOX containing 3 µg/mL DOX induced a significantly higher apoptosis rate in B16 cells (56.1%) than LEVs alone (0.21%, *p* < 0.0001) or DOX alone (20.1%, *p* < 0.0001). These results further confirmed that LEV-DOX has a marked advantage over DOX alone in promoting apoptosis. This effect is likely due to LEV’s ability to increase intracellular drug concentration for enhanced drug cytotoxicity, by either increasing its delivery or facilitating its accumulation, enabling DOX to kill tumor cells more effectively and in a shorter time frame.

### 3.4. Effects of LEVs on ROS and ICD Activity Induced by DOX

**Figure 5:**
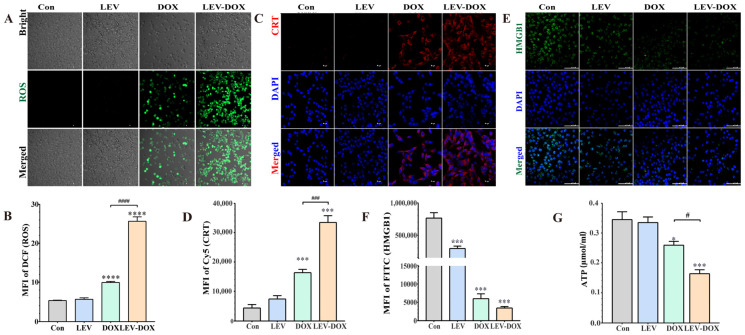
Evaluation of ROS and ICD-associated DAMP levels in B16 cells at 6 h post-treatment with LEVs, free DOX or LEV-DOX. Production of ROS in B16 cells was measured by the MFI of 2′,7′-dichlorofluorescein (DCF) by laser confocal microscope (**A**) and flow cytometry (**B**). Results showed weak ROS signals in untreated and LEV-treated B16 cells, whereas free DOX and LEV-DOX both induced a significant increase in intracellular ROS levels in B16 cells, and LEV loading potentiated DOX-induced ROS production. The expression of CRT (**C**,**D**) on the surface of B16 cells and intracellular HMGB1 (**E**,**F**) and ATP (**G**) was detected by laser confocal scanning microscopy and flow cytometry, respectively, after treatment with LEVs, free DOX and LEV-DOX. Unlike PBS and LEV treatments, DOX significantly increased the number of CRT on the surface of B16 cells and promoted the release of intracellular HMGB1 and ATP, while LEV loading enhanced the effect of DOX-induced ICD. Data are from three independent experiments and analyzed by ANOVA. The error bars are shown as mean ± SEM. * *p* < 0.05, *** *p* < 0.001 and **** *p* < 0.0001, compared with untreated B16 cells (control). # *p* < 0.05, ### *p* < 0.001 and #### *p* < 0.0001, compared with each other. ROS: reactive oxygen species; ICD: immunogenic cell death; DAMPs: damage-associated molecular patterns; LEV: *Lactococcus lactis*-derived extracellular vesicle; LEV-DOX: LEV loaded with doxorubicin; CRT: calreticulin; HMGB1: high mobility group box 1 protein.

Since DOX exhibits multiple anticancer activities, including ROS production for apoptosis, and ICD to release cell surface-exposed calreticulin (CRT) and high mobility group box 1 protein (HMGB1) for tumor-specific immune responses, we first examined whether the LEVs could increase DOX-mediated ROS production after their uptake by tumor cells. Figure 5A,B show that cells treated with PBS or LEVs exhibited almost negligible levels of intracellular ROS, represented by the green fluorescence signal. In contrast, the DOX-treated group displayed a significantly enhanced green fluorescence signal as ROS production in tumor cells. Notably, the green fluorescence signal in the LEV-DOX group was significantly higher than that in the DOX group, suggesting that LEVs substantially enhanced DOX-induced ROS levels. Therefore, LEVs may facilitate the anticancer effect of DOX by increasing intracellular DOX concentration to elicit stronger oxidative stress responses. Next, DOX-mediated ICD in tumor cells was investigated. Figure 5C–F illustrated the expression of CRT on the cell surface and the presence of HMGB1 and ATP in the cytoplasm of B16 cells following 6 h of co-incubation with LEV, DOX, and LEV-DOX. Unlike PBS- and LEV-treated tumor cells, DOX treatment significantly increased the number of CRT molecules on the surface of B16 cells (Figure 5C,D) and promoted the release of HMGB1 (Figure 5E,F) and ATP (Figure 5G) from their cytoplasm, confirming the effect of DOX on ICD. Interestingly, compared to the DOX-treated tumor cells, LEV-DOX treatment not only significantly increased the number of CRT molecules on the cell surface but also markedly reduced intracellular levels of HMGB1 and ATP, indicating a stronger capacity to induce ICD.

### 3.5. Tissue Distribution of LEVs and Its Effect on the Retention Time of DOX In Vivo Following Systemic Administration

**Figure 6:**
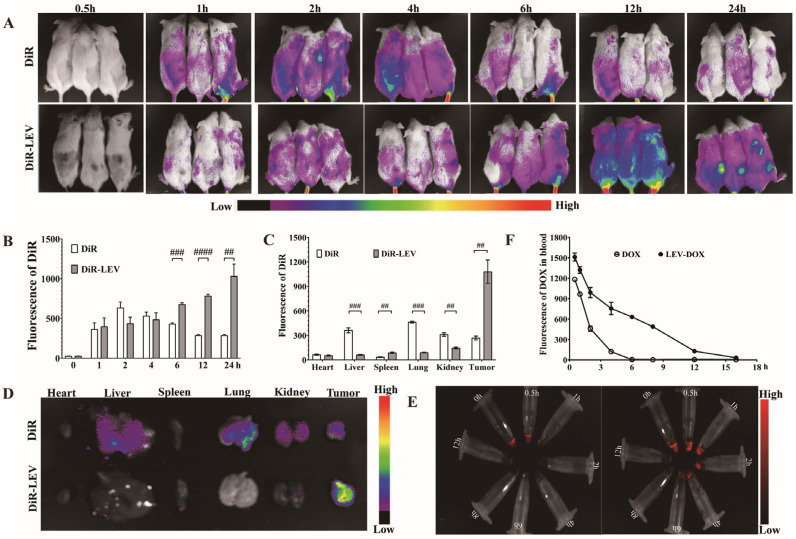
In vivo biodistribution and circulatory stability. (**A**) Representative real-time fluorescence imaging of 4T1 tumor-bearing mice following intravenous injection of DiR or DiR-LEV. (**B**) Mean fluorescence intensity of tumor sites at various time points. (**C**) The cumulative DiR or DiR-LEV in heart, liver, spleen, lung, kidney and tumor at 24 h post-injection by the fluorescence of DiR. (**D**) The fluorescence images of the major organs after intravenous injection of DiR or DiR-LEV for 24 h. (**E**) The fluorescence images of the blood after intravenous injection of DOX or LEV-DOX for 0, 0.5, 1, 2, 4, 6, 8, and 12 h. (**F**) The circulatory stability of free DOX and LEV-DOX by the fluorescence of DOX. ## *p* < 0.01, ### *p* < 0.001, and #### *p* < 0.0001, compared with each other. LEV: *Lactococcus lactis*-derived extracellular vesicle; LEV-DOX: LEV loaded with doxorubicin.

The tumor-targeting ability of LEVs was evaluated by comparing the in vivo distribution of free DiR and DiR-LEV in mice at various time points. Drug distribution and concentrations in different organs (heart, liver, spleen, lung, kidney, and tumor) were analyzed at 0, 0.5, 1, 2, 4, 12, and 24 h post-administration. As shown in Figure 6A,B, the fluorescence intensity of both formulations was very low at 0 h. Over time, DiR-LEV accumulated significantly at the tumor site. Notably, the fluorescence intensity of DiR-LEV in the tumor region was markedly higher than that of free DiR from 12 to 24 h. Figure 6C,D show that free DiR was widely distributed throughout the body, with significant accumulation in the liver, lung, and spleen, but relatively weak fluorescence at the tumor site, indicating that free DiR lacks tumor-targeting ability. In contrast, DiR-LEV was highly concentrated in tumor tissues with minimal presence in other organs. These results demonstrate that LEV possesses strong tumor-targeting capabilities.

To determine whether LEVs can increase the overall retention time of DOX in vivo, we measured plasma fluorescence intensity of the colored DOX at 0, 0.5, 1, 2, 4, 6, 8, and 12 h after intravenous injection of DOX and LEV-DOX into mice. Figure 6C,D showed that the fluorescence intensity of free DOX in the blood decreased rapidly over time, with a plasma half-life of only 1.14 h. In contrast, the plasma half-life of LEV-DOX was 4.64 h, much higher than that of free DOX, indicating the retention capacity of the LEV nanoparticles in vivo, having the potential to produce a more sustained pharmacological effect.

### 3.6. Anti-Tumor Efficacy of LEVs and LEV-DOX in a Subcutaneous Xenograft Tumor Model

**Figure 7:**
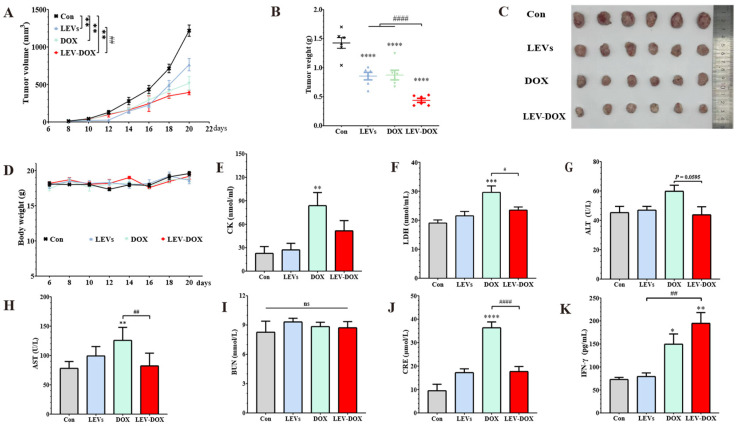
Anti-tumor effect in vivo and blood biochemistry. Mouse melanoma B16 cells were subcutaneously injected into the right flank of C57BL/6 mice. (**A**) Average tumor-growth curves of B16 tumor-bearing mice with different treatments as indicated. (**B**) After the experiment, the mice were euthanized and the tumor was weighed; (**C**) images of the tumor dissected after mouse sacrifice. (**D**) Body weights from four groups of B16 tumor-bearing mice during treatment. (**E**–**K**) Blood biochemistry data of mice on the third day after the last treatment. Data are from six independent experiments and analyzed by ANOVA. The error bars are shown as mean ± SEM. * *p* < 0.05, ** *p* < 0.01, *** *p* < 0.001 and **** *p* < 0.0001 compared with PBS-treated group (Con); # *p* < 0.05, ## *p* < 0.01 and #### *p* < 0.0001 compared with each other. LEV: *Lactococcus lactis*-derived extracellular vesicle; LEV-DOX: LEV loaded with doxorubicin.

**Figure A2:**
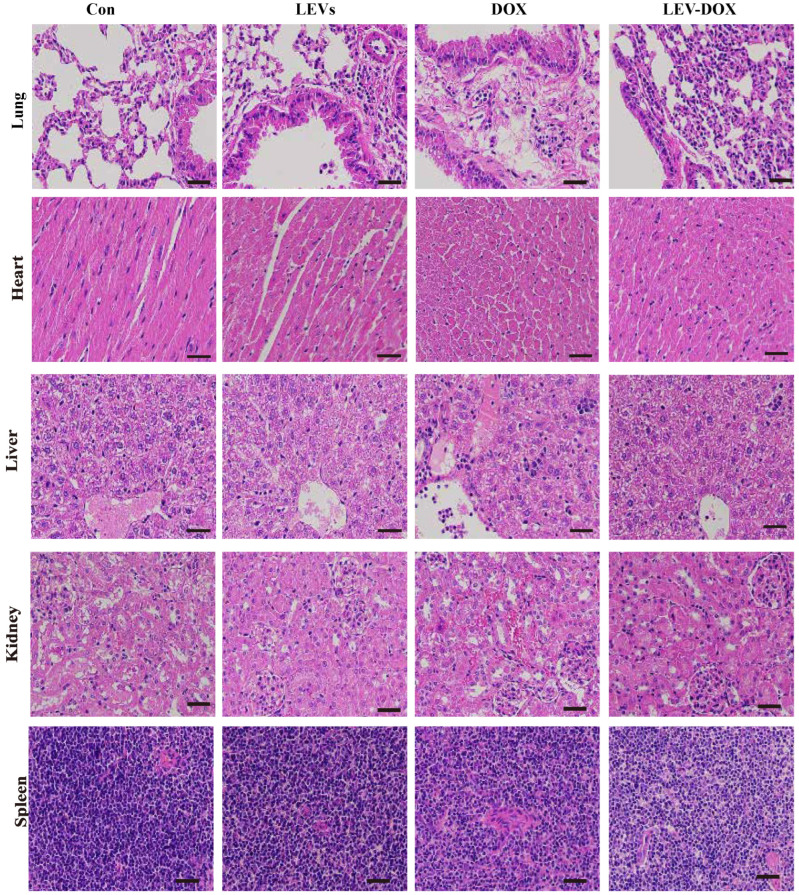
Representative images of H&E-stained sections of the primary organs. Bar = 50 μm.

Finally, we wanted to evaluate the efficacy of the probiotic LEV-encapsulated DOX on tumor growth in vivo. To this end, we established a subcutaneous xenograft tumor model using B16 melanoma cells to compare the inhibitory effects of systemically administered LEV, DOX, and LEV-DOX on tumor cell proliferation. When the subcutaneous tumor volume reached approximately 10 mm^3^, tumor-bearing mice were randomly assigned to four treatment groups: control (PBS), LEVs (10 mg/kg), DOX (2 mg/kg), and LEV-DOX (LEV: DOX = 10 mg/kg: 2 mg/kg). Treatments were administered every three days for a total of four doses. We found that LEV, DOX and LEV-DOX significantly inhibited the growth rate of B16 xenograft tumors, with tumor weights markedly lower than those in the PBS group at day 18. Encapsulation of DOX within LEVs significantly enhanced its anti-tumor efficacy compared to DOX alone (Figure 7A–C). Although none of the treatment groups significantly affected the body weight of the mice at the administered doses (Figure 7D), serum biochemical markers revealed that 2 mg/kg DOX significantly elevated levels of creatine kinase (Figure 7E), lactate dehydrogenase (Figure 7F), aspartate aminotransferase (Figure 7G), blood urea nitrogen (Figure 7H) and creatinine (Figure 7J). In contrast, 10 mg/kg LEVs did not significantly alter these cardiac, hepatic, or renal function markers and even attenuated their elevations induced by DOX (Figure 7E–J). Furthermore, histopathological analysis corroborated these findings: while DOX treatment caused severe inflammatory damage and fibrosis in these tissues, along with renal tubular atrophy and renal arteriosclerosis, only mild inflammatory cell infiltration was observed in the cardiac, hepatic, and renal tissues of the LEVs and control groups. Notably, LEV-DOX treatment mitigated the DOX-induced inflammatory damage and fibrosis (Figure A2). Additionally, both DOX- and LEV-DOX-treated groups had significantly increased serum levels of the cytokine IFN-γ (Figure 7K), although the difference between LEV-DOX and DOX alone was not significant.

### 3.7. Effects of LEVs on Immune Cells in Draining Lymph Nodes and Tumor Tissues In Vivo

**Figure 8:**
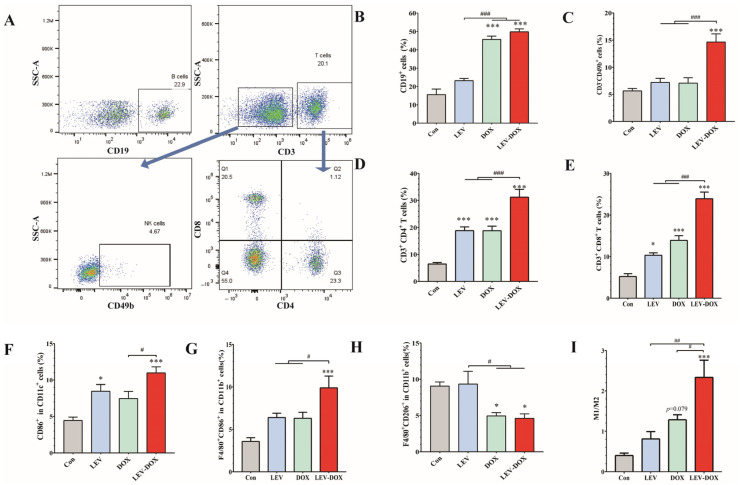
Immune cell expression detected by flow cytometry in mouse lymph nodes. (**A**) Schematic diagram of T, B and NK cell circle gates. The frequencies of B cells (**B**), NK cells (**C**), CD4^+^T cells (**D**), CD8^+^T cells (**E**), DCs (**F**), M1 macrophages (**G**) and M2 macrophages (**H**) in draining lymph nodes. (**I**) Ratio of M1 cells to M2 cells. Data are from six independent experiments and analyzed by ANOVA. The error bars are shown as mean ± SEM. * *p* < 0.05 and *** *p* < 0.001 compared with the untreated group (Con); # *p* < 0.05, ## *p* < 0.01, and ### *p* < 0.001 compared with each other. LEV: *Lactococcus lactis*-derived extracellular vesicle; LEV-DOX: LEV loaded with doxorubicin.

**Figure A3:**
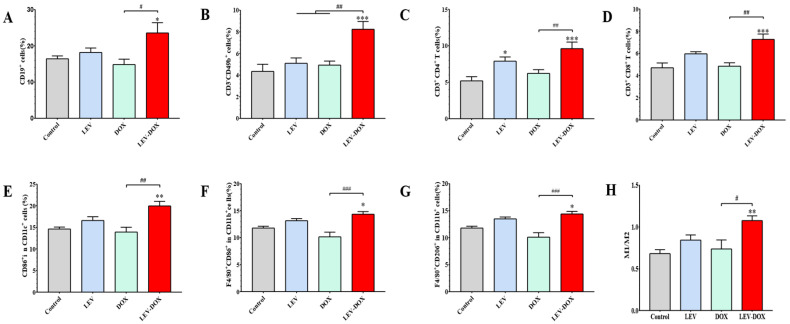
Immune cell expression detected by flow cytometry in mouse spleen. (**A**) The frequencies of B cells, NK cells (**B**), CD4^+^ T cells (**C**), CD8^+^ T cells (**D**), DCs (**E**), M1 macrophages (**F**) and M2 macrophages (**G**) in draining spleen. (**H**). Ratio of M1 cells to M2 cells. Data are from six independent experiments and analyzed by ANOVA. The error bars are shown as mean ± SEM. * *p* < 0.05, ** *p* < 0.01, and *** *p* < 0.001 compared with the untreated group (Con); # *p* < 0.05, ## *p* < 0.01, and ### *p* < 0.001 compared with each other.

Based on the nontoxic immune stimulating and passive tumor-targeting capability of the probiotic LEVs and the ICD function of DOX, we next examined the impact of LEV-DOX on immune cells in vivo following systemic administration by flow cytometric analysis of tumor-draining lymph nodes (TDLNs) and spleens of mice. Figure 8 shows that LEVs increased the proportions of DCs (Figure 8F), CD4^+^ T cells (Figure 8D), and CD8^+^ T cells (Figure 8E) in the lymph nodes, whereas DOX increased the proportions of B cells (Figure 8B), CD4^+^ T cells, and CD8^+^ T cells in the lymph nodes and decreased the proportion of M2 macrophages (Figure 8H). Compared with DOX or LEVs alone, intravenous administration of LEV-DOX enhanced the numbers of B cells, natural killer (NK) cells (Figure 8C), CD4^+^ T cells, CD8^+^ T cells, DCs, and M1 macrophages, but decreased the number of M2 macrophages in the draining lymph nodes. These results indicated that first, the probiotic LEVs, as an adjuvant, can increase the proportions of DCs, CD4^+^ T cells, and CD8^+^ T cells in the lymph nodes, thereby exerting a strong anti-tumor immunity. Second, after encapsulating DOX with ICD activity, LEV-DOX not only promoted the innate anti-tumor immune responses, represented by the increases in NK cells and M1 macrophages, but also enhanced the adaptive anti-tumor immune response, in terms of elevated numbers of B cells, DCs, CD4^+^ T cells, and CD8^+^ T cells. The proportions of immune cells in the spleen also showed that intravenous injection of LEV-DOX significantly increased the proportions of innate immune cells such as NK cells and M1 macrophages, as well as adaptive immune cells including B cells, DCs, CD4^+^ T cells, and CD8^+^ T cells, potentially enhancing the Th1-type immune response and anti-tumor effect (Figure A3).

**Figure 9:**
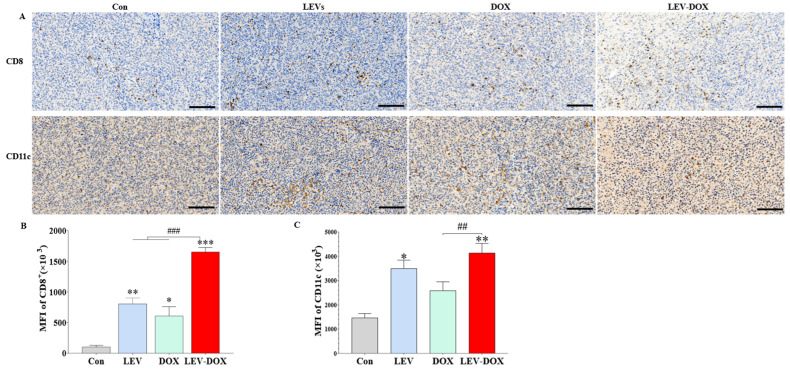
Immune cell infiltration of tumors in mice. (**A**) Immunohistochemical staining of tumor tissues from tumor-bearing mice under different treatments. Stains include CD8 (the upper layer of (**A**)) and CD11c (the lower layer of (**A**)), with corresponding quantitative analyses (**B**,**C**). Bar = 50 μm. Data are from three independent experiments and analyzed by ANOVA. The error bars are shown as mean ± SEM. * *p* < 0.05, ** *p* < 0.01, and *** *p* < 0.001 compared with the PBS group (Con); ## *p* < 0.01, and ### *p* < 0.001 compared with each other. LEV: *Lactococcus lactis*-derived extracellular vesicle; LEV-DOX: LEV loaded with doxorubicin.

One of the primary challenges limiting vaccine efficacy is the immunosuppressive and hypoxic TME, which impairs the immune system’s ability to mount a robust anti-tumor response, during which DC and CD8^+^T cells play important roles. Therefore, we investigated the effects of LEVs and LEV-DOX on the attraction of mature DCs and CD8^+^ T cells in tumor tissue following systemic administration. Figure 9 showed that, in the tumor tissues of PBS-treated mice, there was minimal infiltration of CD8^+^ T cells and CD11c^+^ DCs, indicating a weak immune response within the tumor microenvironment. In the group treated with LEVs alone, however, infiltration of CD8^+^ T cells and CD11c^+^ DCs was significantly higher than that in the PBS-treated control group, demonstrating LEV’s ability to activate immune responses in the TME. Interestingly, the LEV-DOX groups resulted in significantly greater infiltration of CD8^+^ T cells, and DCs, compared with the PBS and DOX-only groups, indicating that LEVs promoted the accumulation of inflammatory immune cells in tumor tissues, which facilitate the capture of the tumor antigen and present the tumor antigen for anti-tumor immune responses.

## 4. Discussion

Unlike traditional tumor vaccines, ISVs represent a distinct category of tumor vaccines that contain no exogenous tumor antigen, but immune activators to condition the local APCs in the immune-suppressive TME to take up and present endogenous tumor antigens, greatly minimizing the systematic immune-related adverse reactions. However, this approach is not suitable for intracorporal tumors or late-stage disseminated tumors. In recent years, nanomaterials have emerged as powerful tools in ISVs due to their ability to passively target tumor tissues over a long distance via the EPR effect, encapsulate anti-tumor drug substances, and modulate the TME [20,21]. Currently, substances used for the preparation of nanomaterials primarily include natural derivatives such as virus-like particles or bacterial extracellular vesicles, inorganic materials such as gold or silica, liposomes, polymer-based materials like polyethyleneimine, natural polycationic chitosan, and organic/inorganic hybrid materials. However, these materials face key challenges related to reproducibility, scalability, and safety [20]. Compared with other nanomaterials, BEVs offer several advantages, including strong immunostimulatory effects, non-replicability, ease of modification, cargo protection, targeted delivery to tumors via the EPR effect, and higher safety. Nonetheless, BEVs also have disadvantages, such as uncontrollable composition, susceptibility to immune clearance, the lack of standardized large-scale production methods, and most importantly, residual bacterial toxins [22], which remain a major barrier to the application of bacteria or their derivatives. For example, outer membrane vesicles (OMVs) secreted by the outer membrane of Gram-negative bacteria, although widely used as nanocarriers for anticancer immunotherapy, pose significant safety and regulatory challenges due to the high LPS contents in OMVs [23]. In this study, we used extracellular vesicles released by probiotic *Lactococcus lactis* MG1363, which is GRAS, as a delivery carrier and immune activator. This bacterium, lacking LPS and significant virulence genes, has been employed as a cellular factory for various heterologous products, and a mutant strain expressing the cytokine interleukin-10 has been used in clinical trials for patients with inflammatory bowel disease [15]. Although *Lactococcus lactis*, a Gram-positive bacterium, naturally releases fewer LEVs, the yield of LEVs can be significantly increased by adjusting culture conditions such as pH, bacterial growth time, and broth concentration [20]. For example, the yield of LEVs released by *Lactococcus lactis* was increased more than tenfold by adding lysozyme and ampicillin, which weaken the cell walls, to the culture medium.

In the present study, our LEVs retained in the supernatant of *Lactococcus lactis* culture after 100 kD ultrafiltration exhibited a particle size of 131.1 ± 3.94 nm. LEV-DOX particles measured 184.6 ± 2.05 nm, consistent with the hypothesis that nanoparticles sized between 100 and 200 nm have prolonged plasma circulation times and enhanced tumor tissue accumulation [24]. The plasma retention time and tissue distribution of intravenously administered LEVs further supported this hypothesis (Figure 6). LEVs increased the plasma half-life of the chemotherapeutic drug DOX by more than fourfold. Twelve hours post-intravenous administration, LEV concentrations in tumor tissues were significantly higher than in other tissues, indicating effective plasma retention and tumor targeting. Most importantly, we found that the probiotic LEVs were taken up more easily by tumor cells and macrophages than by normal cells (Figure 3B), and their encapsulated drug DOX tended to be released at the lower pH of the tumor environment rather than the normal pH of physiological conditions (Figure 1G). This may be because the phosphatidylethanolamine (PE) and phosphatidylserine (PS) components in the *Lactococcus lactis* cell membrane are similar to those in tumor cell membranes [25]. Additionally, our results further confirm that DOX remains encapsulated within liposomes or extracellular vesicles at physiological pH, while the drug is gradually released from liposomes in an acidic environment [26]. Finally, intravenous administration of LEVs did not adversely affect body weight or the function of organs such as the heart, liver, and kidneys in mice, suggesting that our newly generated probiotic LEVs represent a relatively safe tumor-targeting nano delivery system.

Cellular uptake and exocytosis are critical determinants of delivery efficiency and biosafety for drug delivery systems. Both synthetic nanoparticles and extracellular vesicles (EVs) enter cells via endocytosis, traffic through the endosomal pathway, and undergo exocytosis for clearance, but their intracellular behaviors differ significantly. Nanoparticles are mainly internalized through non-specific endocytosis with limited endosomal escape, and their exocytosis is strongly regulated by surface physicochemical properties. As reported by Ho et al., low alkylation modification directs gold nanoparticles toward efficient exocytosis via the EV pathway, whereas high hydrophobicity favors lysosomal trapping and intracellular accumulation, increasing toxicity risks [27]. In comparison, EVs are taken up through specific membrane recognition and achieve efficient cytosolic delivery via membrane fusion. They follow natural physiological vesicle secretion routes for exocytosis, leading to mild intracellular retention and favorable biocompatibility [28]. Given these features, EVs hold distinct advantages over synthetic nanoparticles in long-term safety and in vivo translation, while nanoparticle exocytosis mechanisms remain highly dependent on surface engineering strategies [29].

Common strategies to stimulate the generation of endogenous tumor antigens include using chemical and physical therapies to induce ICD in the tumor cells, which not only promotes the release of endogenous tumor antigens but also triggers the release of damage-associated molecular patterns (DAMPs), such as CRT, ATP, HSP70/HSP90, and HMGB1. These DAMPs recruit APCs to the tumor tissue, facilitate phagocytosis of the tumor antigens, and enhance CTL responses [20,30]. Studies have confirmed that low doses of DOX can induce ICD and upregulate the expression of MHC I and II molecules, thereby enhancing tumor antigenicity [31]. However, when the cumulative dose of DOX exceeds 550 mg/m^2^, it may cause irreversible severe heart failure and damage to other organs such as the brain, liver, and kidneys [18,32]. Although the safety profile of liposome-encapsulated DOX has improved, its efficacy is not superior to that of conventional DOX [33]. This may be due to the highly stable nature of liposomes (e.g., Doxil), which results in poor drug release within tumor tissues [34]. In the current study, we confirm that our probiotic LEV-encapsulated DOX can significantly enhance ROS production and ICD levels in tumor cells in vitro. Furthermore, systemic administration of LEVs significantly inhibited the growth of subcutaneously transplanted B16 tumor cells. We also found that the probiotic LEVs markedly reduced serum biochemical markers indicative of cardiac, hepatic, and renal injury caused by DOX. Histopathological analysis of major organs demonstrated that LEVs mitigated inflammatory damage and fibrosis induced by DOX in cardiac, hepatic, and renal tissues, indicating that LEVs can reduce the side effects of chemotherapeutic drugs through tumor targeting. This effect may be attributed to the tumor-targeting capability of LEV, which promotes the accumulation of DOX in tumor tissues, thereby reducing toxicity to normal tissues while inhibiting the growth of B16 cells.

Our previous studies have demonstrated that *Lactococcus lactis* exhibited adjuvant effects that promote DC maturation and Th1 responses [35]. In the present study, we further found that LEVs from *Lactococcus lactis* could also significantly enhance the expression of DC costimulatory molecules (CD40, CD80, and CD86), major histocompatibility complex (MHC) class I and II molecules, and the secretion of cytokines (IL-12, TNF-α, IL-6, and IL-1β) (Figure 2A,B). In addition, the LEVs also stimulated the expression of the DC surface molecule CCR7 and promoted DC migration (Figure A1). Moreover, when the LEV-activated DCs were co-cultured with splenic lymphocytes in vitro, there was a significant increase in the number of CD4^+^ and CD8^+^ T cells (Figure 2C), consistent with the findings of Lee et al. [17]. In vivo experiments demonstrated that intravenously administered LEVs increased the numbers of DCs, M1 macrophages, CD4^+^ T cells, and CD8^+^ T cells in the draining lymph nodes (Figure 8D–I), as well as the numbers of DCs and CD8^+^ T cells in tumor tissues (Figure 9A–C), significantly inhibiting the growth of transplanted B16 tumor cells. These results indicate that LEVs function not only as carriers for tumor-targeted delivery over long distances but also as potent immune stimulants.

## 5. Conclusions

In this study, we demonstrated that extracellular vesicles derived from *Lactococcus lactis* can act as a systemically administered ISV. Attributed to their intrinsic immunostimulatory capacity and the property of passively accumulating in tumor tissues, these vesicles exert prominent anti-tumor immune effects. Moreover, the surfaces of these nontoxic LEVs are enriched with TLR-agonistic components that readily bind to antigen-presenting cells. Therefore, by leveraging the dual targeting features of these vesicles to both antigen-presenting cells and tumor tissues, they are better suited to deliver DC-recruiting molecules such as Flt3L, T cell stimulants like OX40L or IL-12, or surface-modified maleimide that enhances tumor antigen enrichment. This approach can create efficient cancer vaccine carriers, which could be widely applied as adjuvant therapies before and after surgical treatment of solid tumors.

## Data Availability

The original contributions presented in this study are included in the article. Further inquiries can be directed to the corresponding authors.

## Abbreviations

The following abbreviations are used in this manuscript:

ALT	alanine aminotransferase
AST	aspartate aminotransferase
BCA	bicinchoninic acid
BEVs	bacterial extracellular vesicles
BUN	blood urea nitrogen
CK	creatine kinase
CRE	creatinine
CRT	calreticulin
CTL	cytotoxic T lymphocyte
DAMPs	damage-associated molecular patterns
DCs	dendritic cells
DL	drug-loading efficiency
DLS	dynamic light scattering
DMEM	Dulbecco’s modified Eagle medium
DMSO	dimethyl sulfoxide
DOX	doxorubicin
EE	encapsulation efficiency
FITC	fluorescein isothiocyanate
GM-CSF	granulocyte-macrophage colony-stimulating factor
GRAS	generally regarded as safe
H&E	hematoxylin and eosin
HMGB1	high mobility group box 1 protein
ICD	immunogenic cell death
ISVs	in situ vaccines
LDH	lactate dehydrogenase
LEVs	Lactococcus lactis extracellular vesicles
LPS	lipopolysaccharide
MAMPs	microbe-associated molecular patterns
MLR	mixed lymphocyte reaction
OMVs	outer membrane vesicles
PDI	polydispersity index
PE	phosphatidylethanolamine
PS	phosphatidylserine
ROS	reactive oxygen species
SPF	specific pathogen-free
TAAs	tumor-associated antigens
TEM	transmission electron microscopy
TME	tumor microenvironment
